# Supplementary figures and images for: Structures of vesicular stomatitis virus glycoprotein G alone and bound to a neutralizing antibody
Source: PLoS Pathog. 2025 Oct 27;21(10):e1013589. doi: 10.1371/journal.ppat.1013589 (PMC12574954; doi:10.1371/journal.ppat.1013589)

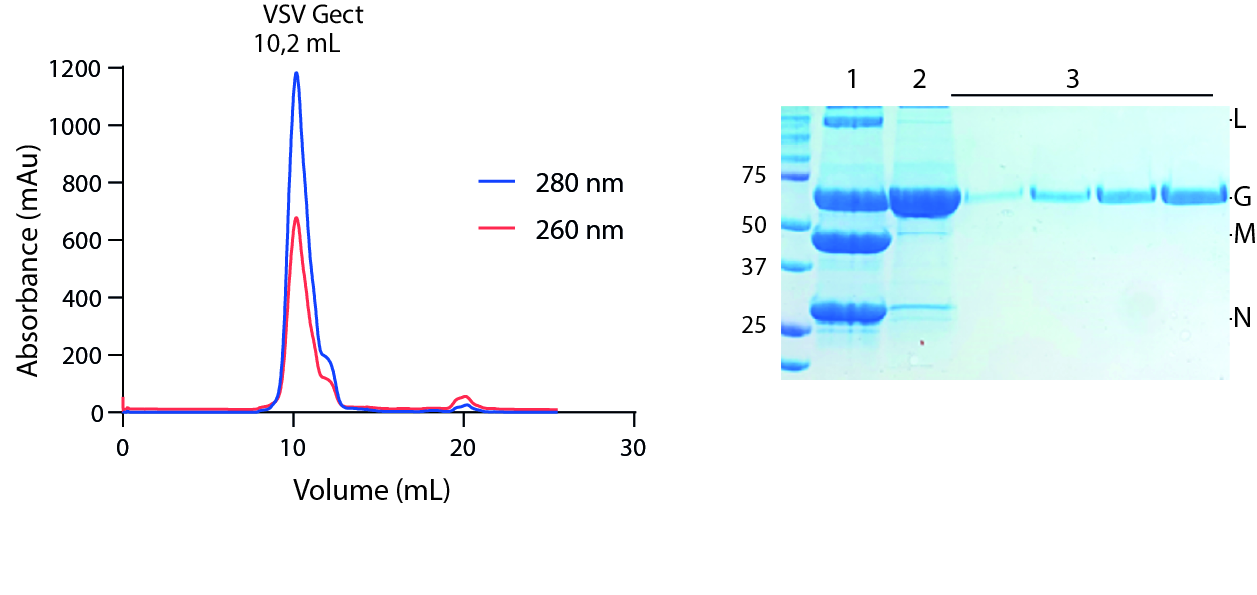

Supplement: S1 Fig — Elution profile of VSV G on a Superdex S200 increase HR 10/300 GL (Cytiva) and Coomassie-stained SDS-PAGE analysis of VSV G purification steps (1: concentrated VSV preparation, 2: VSV G after anion exchange chromatography step, 3: VSV G after size exclusion chromatography step). (TIF) [file ppat.1013589.s001.tif]

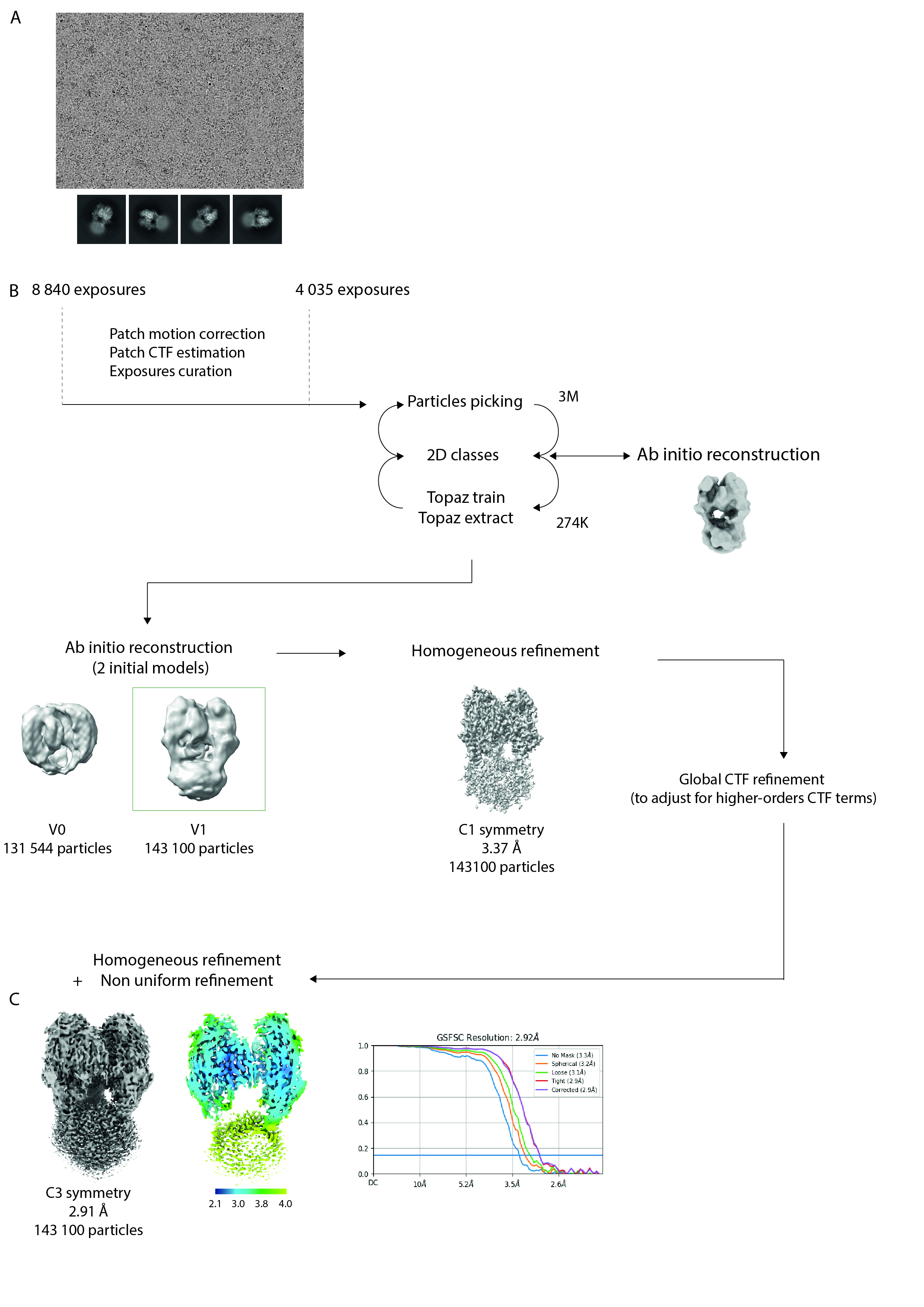

Supplement: S2 Fig — (A) Representative electron micrograph. (B) Cryo-EM data processing workflow in cryoSPARC. (C) Final density used for model building (left) and local resolution map calculated and plotted onto the sharpened VSV G reconstruction. (TIF) [file ppat.1013589.s002.tif]

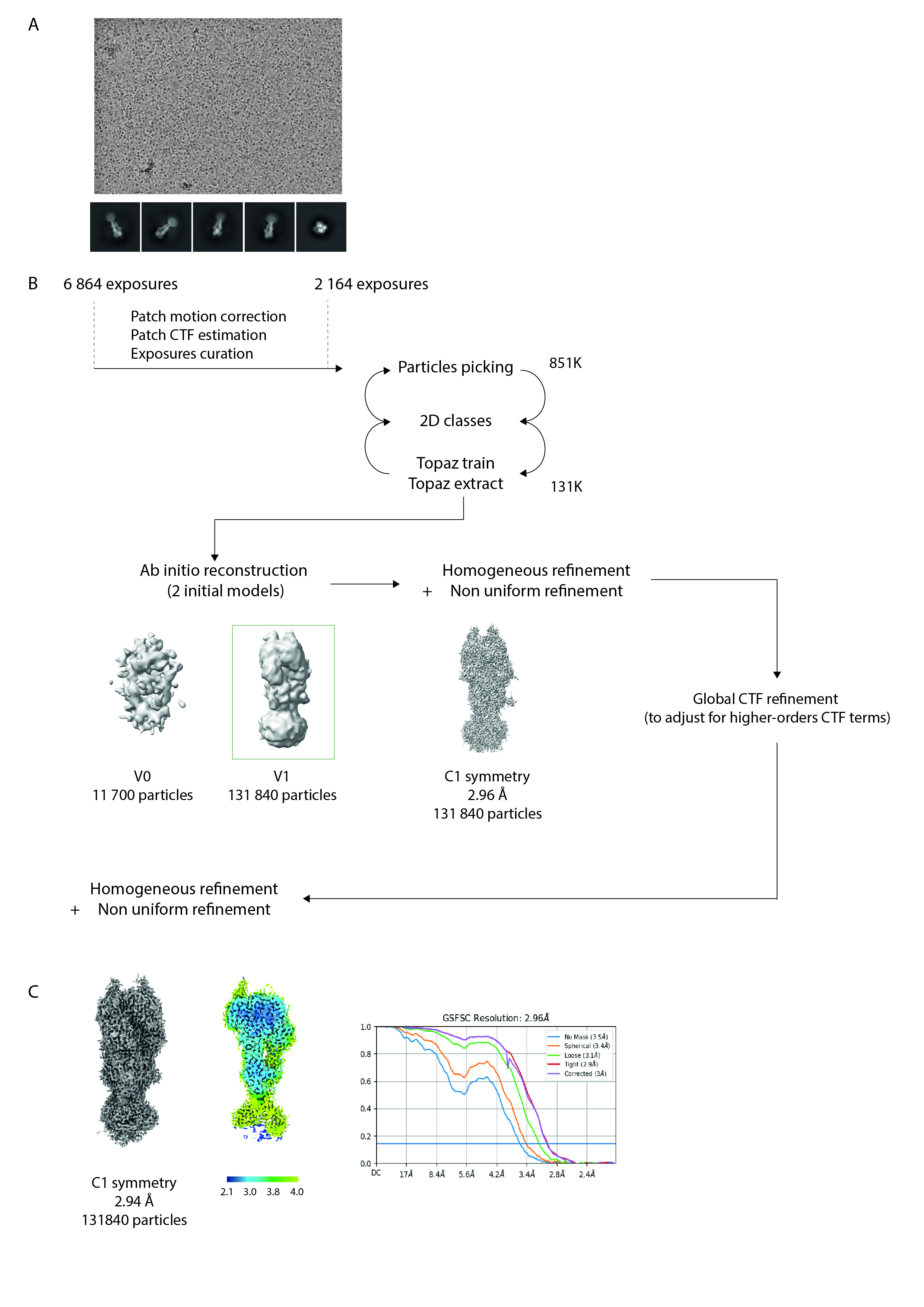

Supplement: S3 Fig — (A) Representative electron micrograph. (B) Cryo-EM data processing workflow in cryoSPARC. (C) Final density used for model building (left) and local resolution map calculated and plotted onto the sharpened VSV G reconstruction. (TIF) [file ppat.1013589.s003.tif]

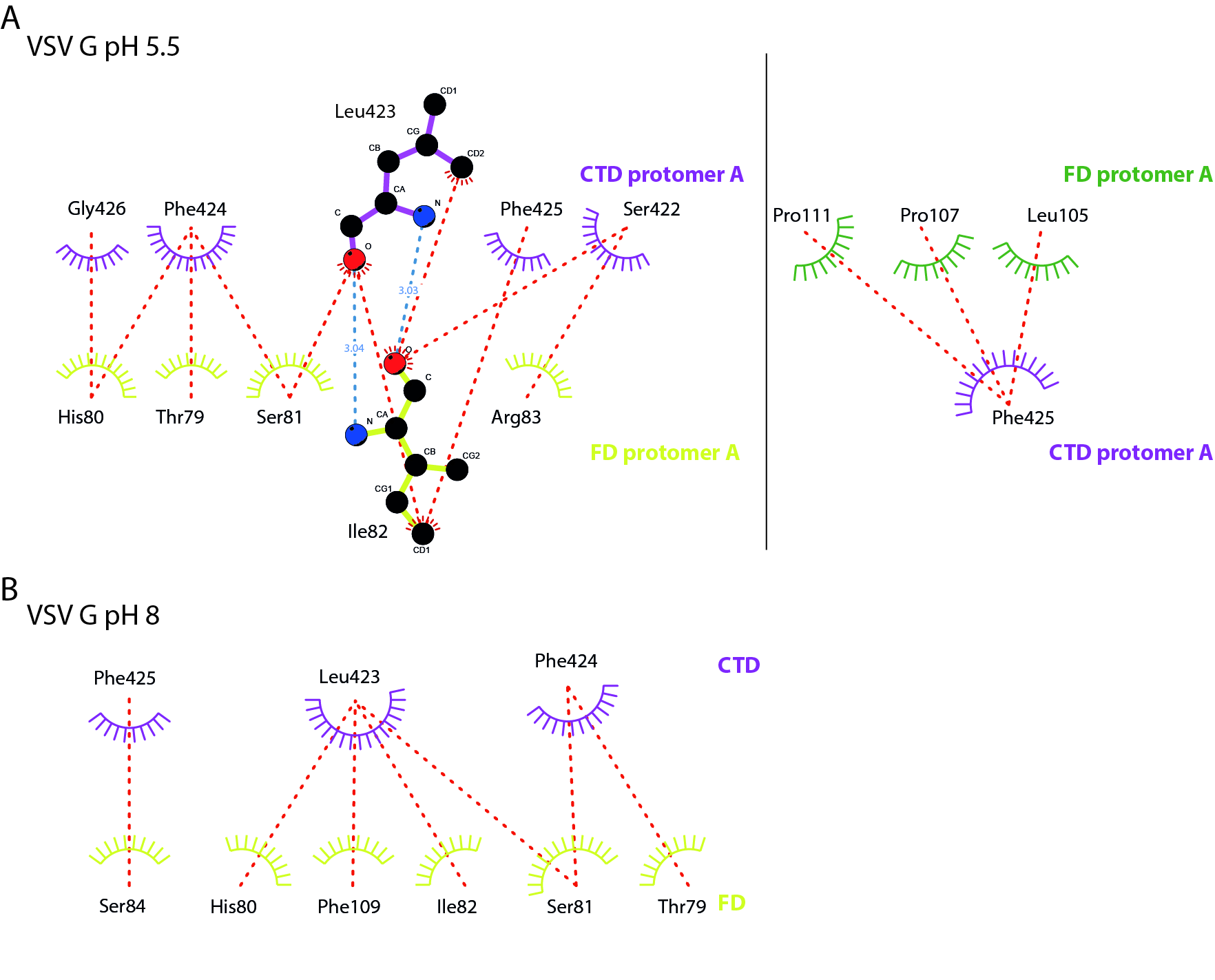

Supplement: S4 Fig — (A) Representation of the interaction of the CTD and the FD at pH 5.5. The left panel shows Dimplot of VSV G CTD (residues 419–426, in magenta) with the FD from protomer A (in yellow), and the right panel shows the Dimplot of the CTD with the FD from protomer B (in green). (B) Dimplot of VSV G CTD with FD at pH 8.0. Putative hydrophobic interactions are depicted in red dashed lines, and polar interactions are in light blue. (TIF) [file ppat.1013589.s004.tif]

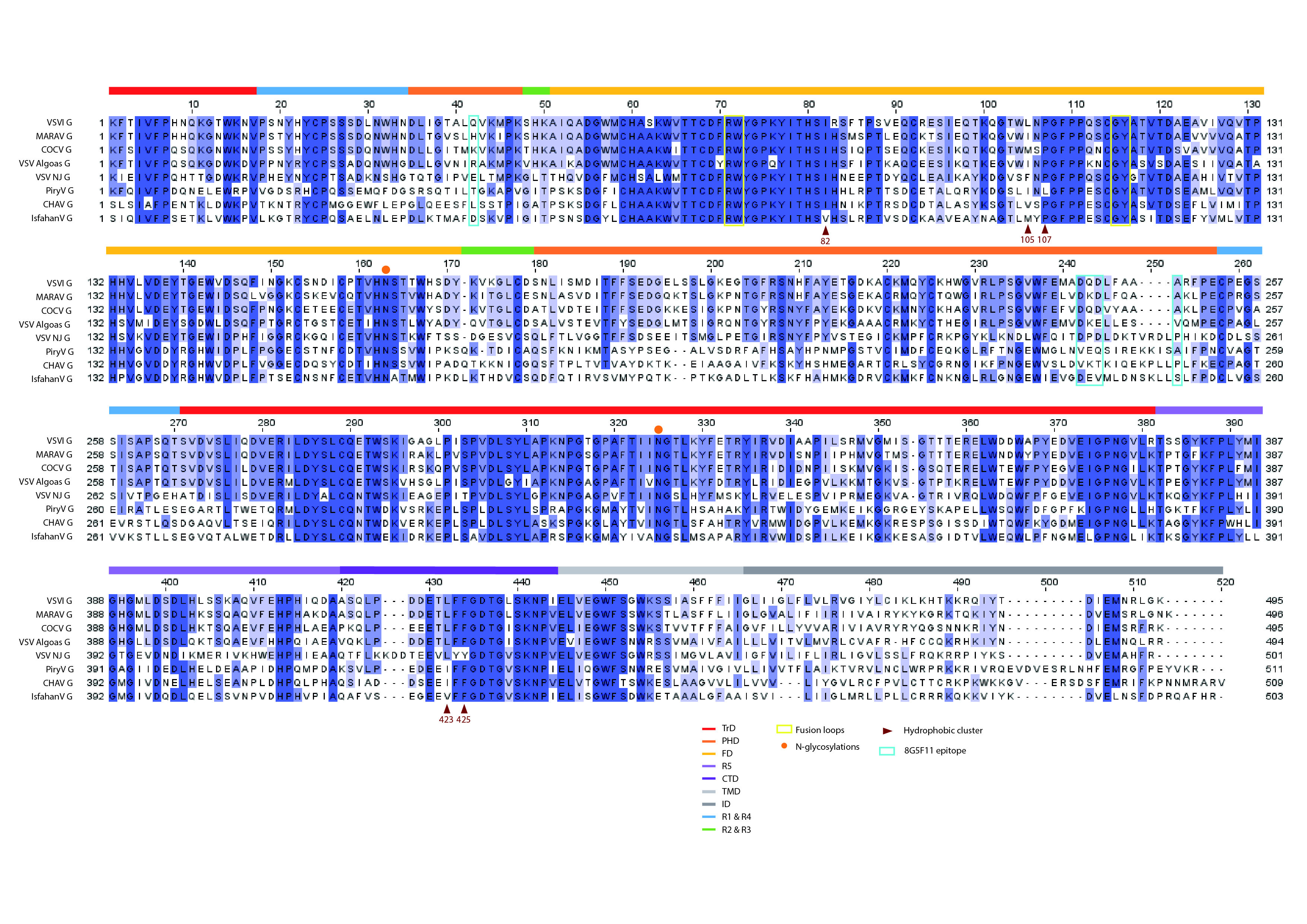

Supplement: S5 Fig — Conserved residues are highlighted in dark blue boxes, while similar residues are shown in lighter blue. VSVI G domains are indicated above the sequence and depicted according to Fig 1A color code. Asparagine carrying N-glycosylation are marked with an orange circle. The residues constituting the fusion loops are framed in yellow boxes. Residues belonging to 8G5F11 epitope are framed in cyan boxes. The residues constituting the hydrophobic patch stabilizing the CTD in the post-fusion conformation are indicated by brown arrows. (TIF) [file ppat.1013589.s005.tif]

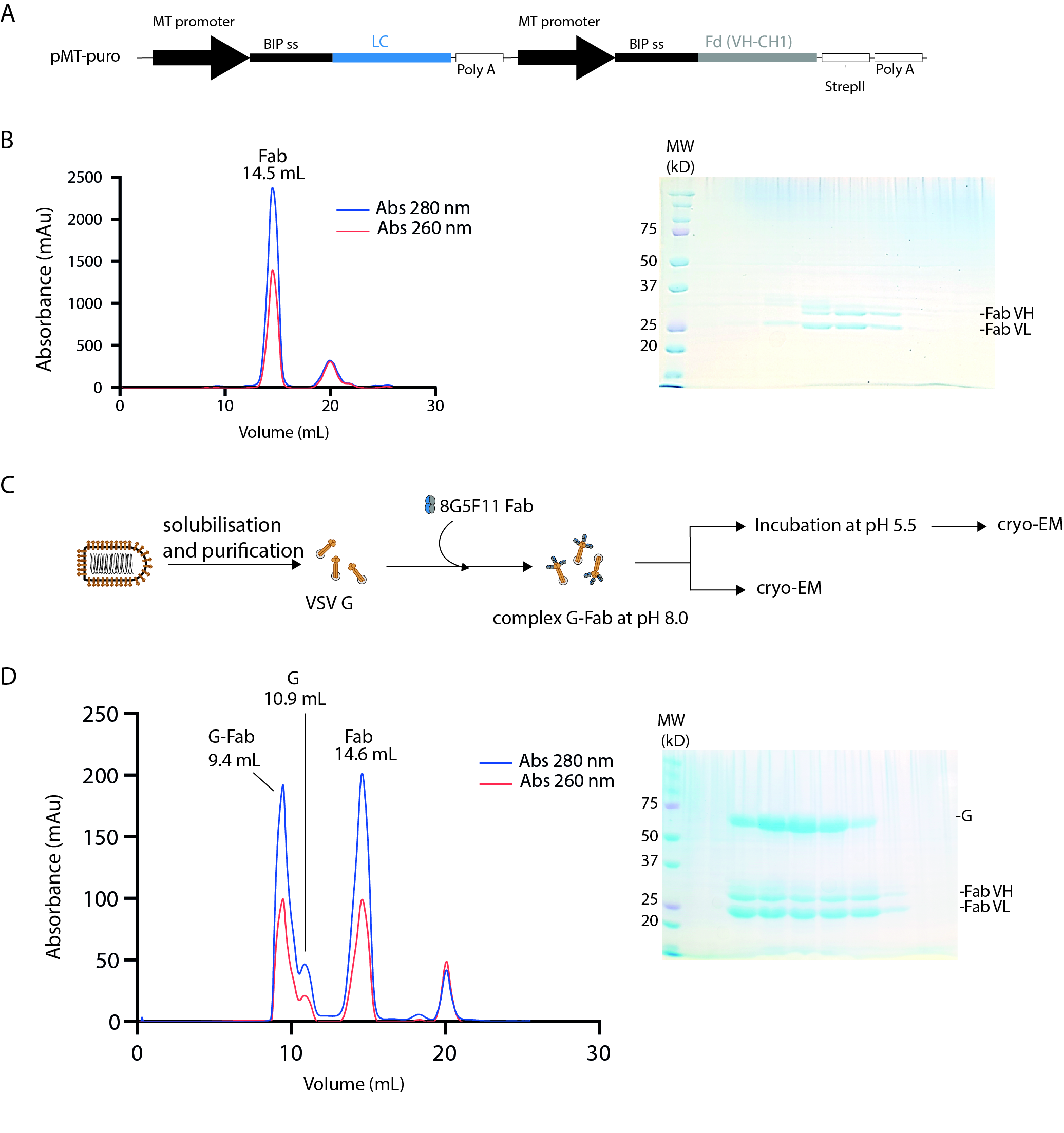

Supplement: S6 Fig — (A) Diagram of the construction used to produce 8G5F11 Fab. (BIP ss = BIP signal sequence; LC = light chain; Fd = part of the heavy chain composing the Fab; StrepII = Strep-tag II). (B) Elution profile of Fab on a Superdex S200 HR 10/300 (Cytiva) in 20 mM Tris-HCl pH 8.0, 150 mM NaCl, 2 mM EDTA (left panel) and Coomassie-stained SDS-PAGE analysis of purified Fab (right panel). (C) Schematic description of VSV G-Fab complex assembly for cryo-EM studies. (D) Elution profile of VSV G-Fab complex on a Superdex S200 HR 10/300 (Cytiva) in 20 mM Tris-HCl pH 8.0, 150 mM NaCl 2 mM EDTA (left panel) and Coomassie-stained SDS-PAGE analysis of purified VSV G-Fab. (TIF) [file ppat.1013589.s006.tif]

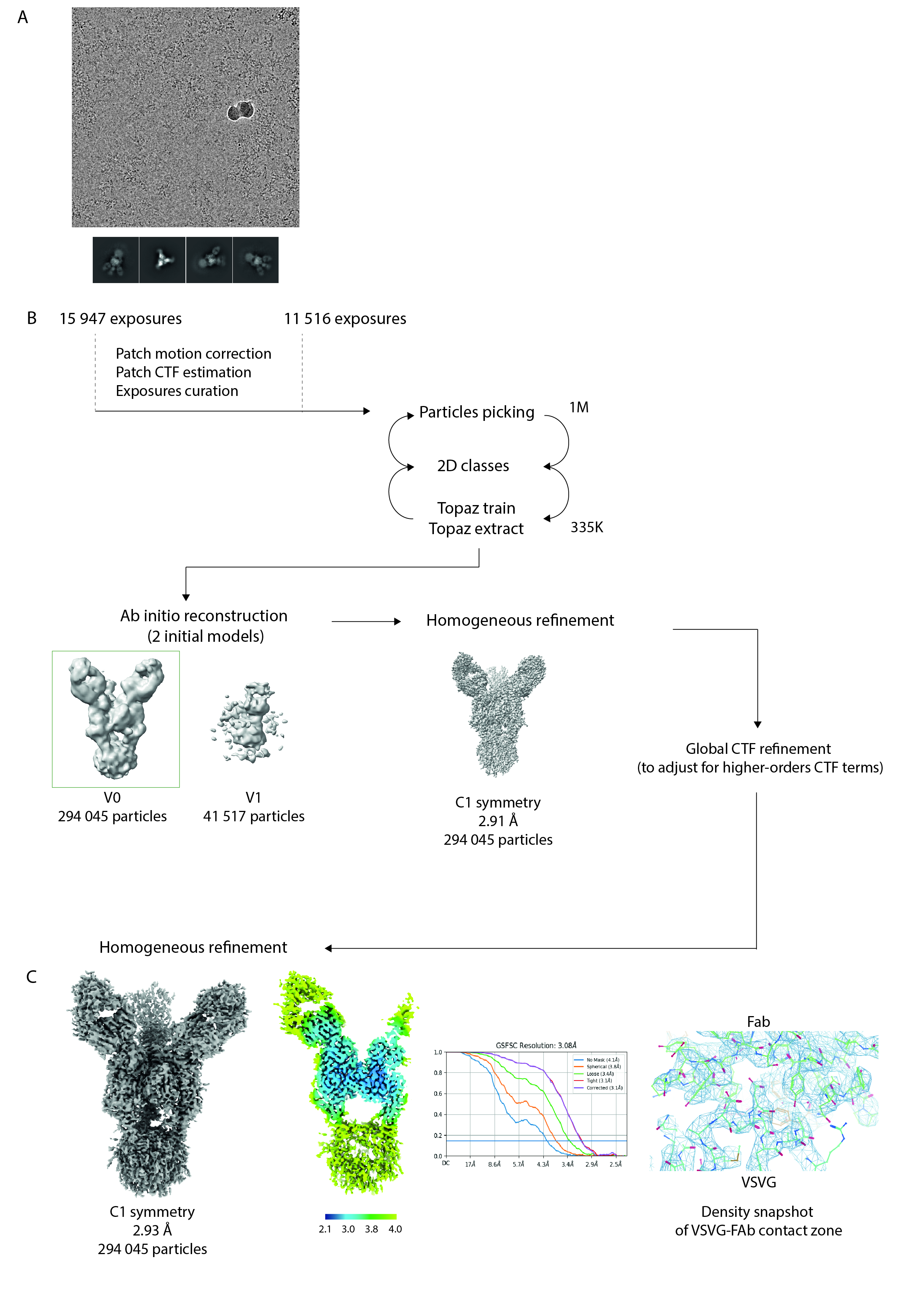

Supplement: S7 Fig — (A) Representative cryo-electron micrograph. (B) Cryo-EM data processing workflow in cryoSPARC. (C) Final density used for model building (left panel) and local resolution map calculated and plotted onto the sharpened VSV G reconstruction (middle panel) and representative fit of atomic model of G and Fab into density (right panel). (TIF) [file ppat.1013589.s007.tif]

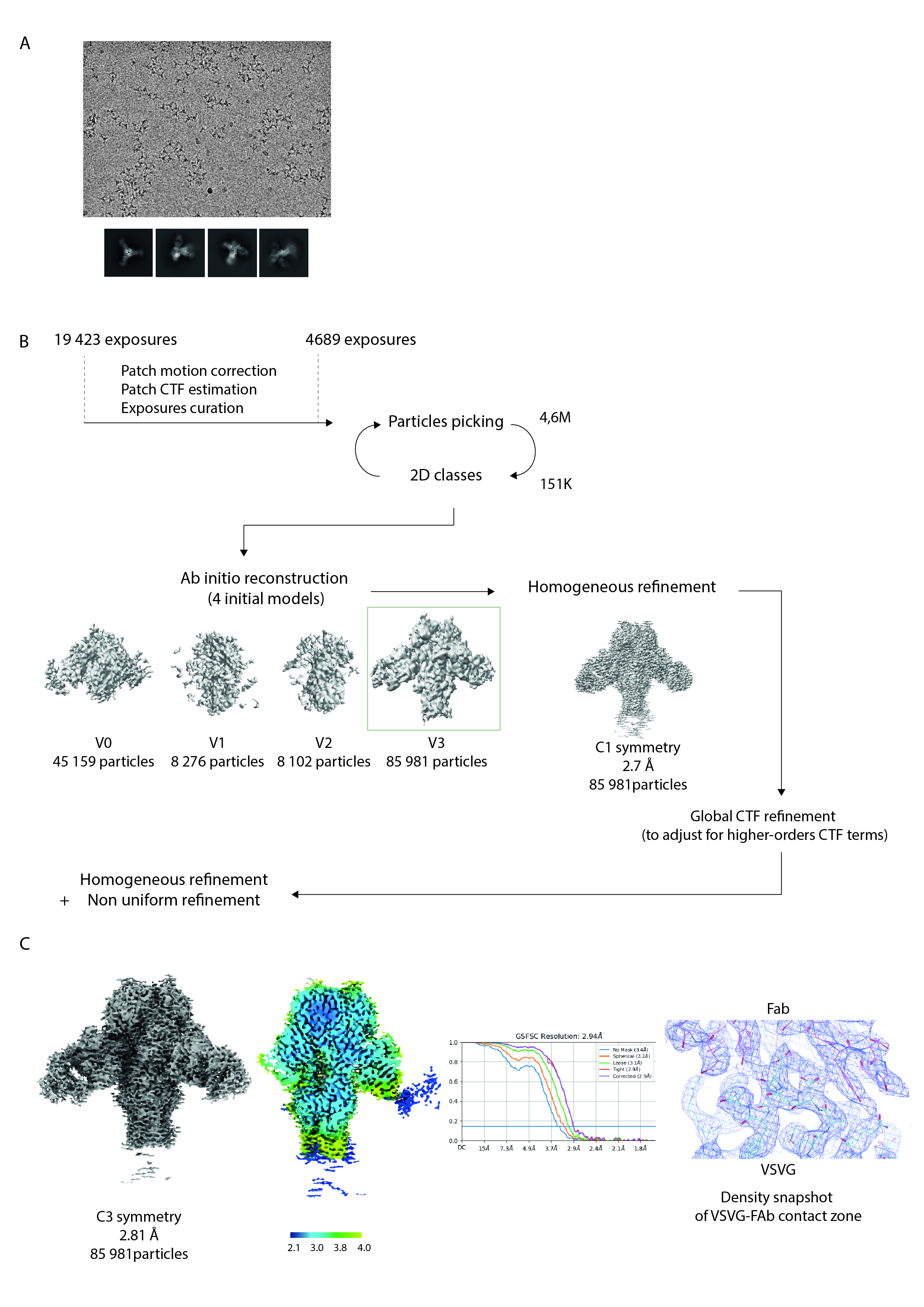

Supplement: S8 Fig — (A) Representative cryo-electron micrograph.(B) Cryo-EM data processing workflow in cryoSPARC. (C) Final density used for model building (left panel) and local resolution map calculated and plotted onto the sharpened VSV G reconstruction (upper right panel) and representative fit of atomic model of G and Fab into density (lower right panel). (TIF) [file ppat.1013589.s008.tif]

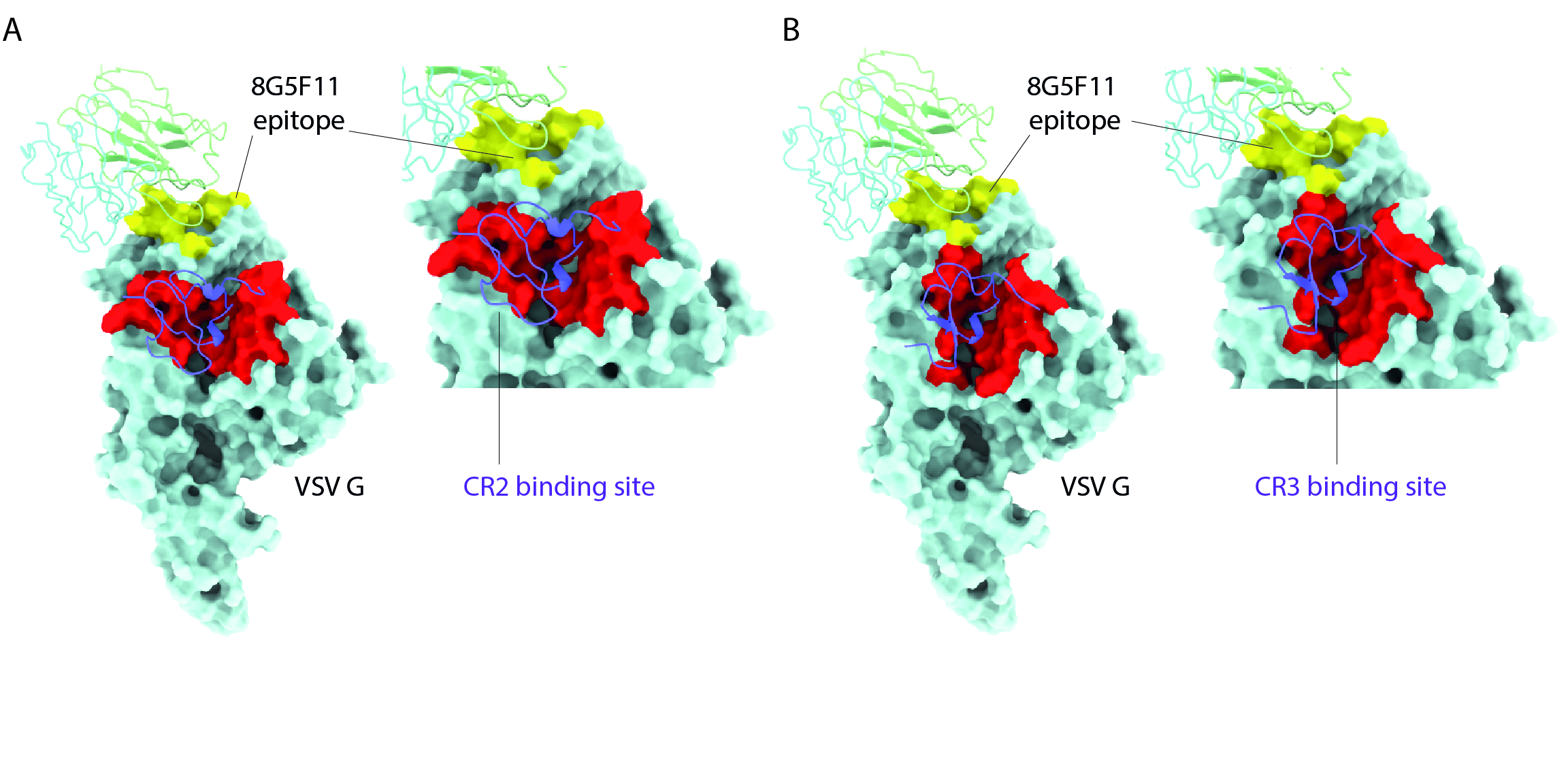

Supplement: S9 Fig — Binding footprint of 8G5F11 and CR2 (A) or CR3 (B) on VSV G. (TIF) [file ppat.1013589.s009.tif]
