# Supplementary material for: Structures of vesicular stomatitis virus glycoprotein G alone and bound to a neutralizing antibody
Source: PLoS Pathog. 2025 Oct 27;21(10):e1013589. doi: 10.1371/journal.ppat.1013589 (PMC12574954; doi:10.1371/journal.ppat.1013589)
Supplement: S2 Table — (DOCX) [file ppat.1013589.s011.docx]

| **Structural domain** | **Number of residues** | RMSD  VSV G post-fusion / 5i2m | RMSD VSV G pre-fusion / 6tit |
| --- | --- | --- | --- |
| **Whole molecule** | 426 | 0,664 (366 Cα) | 0,634 (369 Cα) |
| **FD** | 120  (res 53 to 173) | 0,549 (108 Cα) | 0,500 (99 Cα) |
| **PHD** | 92  (res 35 to 47 and 180 to 260) | 0,492 (88 Cα) | 0,431 (82 Cα) |
| **TrD** | 127  (res 1 to 18 and 272 to 382) | 0,430 (119 Cα) | 0,403 (121 Cα) |
