# Supplementary material for: Structures of vesicular stomatitis virus glycoprotein G alone and bound to a neutralizing antibody
Source: PLoS Pathog. 2025 Oct 27;21(10):e1013589. doi: 10.1371/journal.ppat.1013589 (PMC12574954; doi:10.1371/journal.ppat.1013589)
Supplement: S3 Table — (DOCX) [file ppat.1013589.s012.docx]

| Sensor | Sample | KD (M) | KD Error | ka (1/Ms) | ka Error | kdis (1/s) | kdis Error |
| --- | --- | --- | --- | --- | --- | --- | --- |
| mAb 8G5F11 | Gect pH8.0 | 1,01E-09 | 5,80E-12 | 1,87E+05 | 3,57E+02 | 1,88E-04 | 1,02E-06 |
| Fab 8G5F11 | Gect pH8.0 | 7,17E-10 | 3,18E-12 | 2,39E+05 | 6,64E+02 | 1,72E-04 | 5,94E-07 |
