# Supplementary material for: Structures of vesicular stomatitis virus glycoprotein G alone and bound to a neutralizing antibody
Source: PLoS Pathog. 2025 Oct 27;21(10):e1013589. doi: 10.1371/journal.ppat.1013589 (PMC12574954; doi:10.1371/journal.ppat.1013589)
Supplement: Attachment — Submitted filename: Response to Reviewers.docx [file ppat.1013589.s014.docx]

**Response to Reviewers:**

Reviewer #1: The following experiments would add strength to the hypotheses made by the authors:

1. Lines 150-152: In addition to the pinpointed hydrophobic interactions, highlighting other interactions such as hydrogen bonds and salt bridges (given the prominence of the hydrophilic groove), contributed by the newly resolved CTD region, would allow direct comparison of the bonds required to break and/or formed at the equivalent region during pH-induced structural changes from pre- to post-fusion states. This could be done with detailed supplementary close-up diagrams for the interactions formed at this region or a detailed 2D interaction schematic.

We thank the reviewer for this suggestion. We have modified the orientation of the close-up shown in the inset of figure 2D, which now highlights more clearly the hydrophobic interaction network involving the residues of the CTD in the post fusion conformation.

As requested, we also tried to focus more on polar interactions to complete the previous analysis using 2D plots diagrams calculated by Ligplot+(DIMPLOT). This confirms that the CTD engages mainly hydrophobic interactions -both intra- and inter- protomer at pH 5.5 and intra-protomer at pH 8.0.

We added in supplementary (Fig S4) 2D schematic diagrams to show those interactions between i) the CTD (residues 419-426) and the FD of the same protomer (protomer A) at pH 5.5 ii) the CTD (residues 419-426) and the FD of the neighboring protomer (protomer B) at pH 5.5. iii) the CTD (residues 419-426) and the FD of the same protomer at pH 8.0.

The only polar interaction detected and now listed is between the main chain of the leucine 423 at the level of the CTD with the main chain of isoleucine 82 at the level of the FD of the same protomer at pH 5.5.

2. Lines 391-397: The authors hypothesized a few possible neutralisation mechanisms for mAb 8G5F11, including interfering with VSV-G conformational changes. This should be validated experimentally. As the receptor for VSV, LDL-R, only binds pre-fusion G and does not compete with 8G5F11 binding, the authors could use the receptor (eg. CR3 domain) as conformational probe in a BLI real-time binding experiment to test its binding to Fab-G complexes buffer-exchanged from neutral to acidic pH, and vice versa. Alternatively, the authors could also generate cryoEM 2D classes to observe the effect of pH change on Fab-bound G conformation, if any.

As Munis et al., 2018 (PMID: 30232190) showed that 8G5F11 blocks infection after receptor binding and endocytosis but before genome reverse transcription, the authors could use a membrane fusion assay or a cell-cell fusion assay to test if the mAb interferes with membrane fusion at acidic endosomal pH. The data would further support the BLI/2D averaging experiments above.
Interestingly, in Figure S5 (C), the authors indicated that the G-Fab complex was formed at pH 8.0, followed by acidification to pH 5.5 to obtain the post-fusion G-Fab structure. Does this mean that 8G5F11 does not hinder conformational changes from pre- to post-fusion? This needs to be clarified.

We thank the reviewer for this suggestion. We agree that an experimental validation would strengthen the study. However, in our hands, BLI assays with VSV G at acidic pH resulted in strong non-specific binding signals, making this approach unsuitable for a reliable analysis. Moreover, cryo-EM analyses of Fab-G complexes at multiple pH values would require extensive optimization of sample and data collection pipelines.

Furthermore, in the revised version of our manuscript, we discuss more the work of Munis et al., which indeed showed convincingly that 8G5F11 blocks infection after receptor binding and endocytosis. **Thus, following reviewer suggestion, we performed cell-cell fusion assays to directly assess the effect of 8G5F11 on membrane fusion.** These experiments clearly showed that no fusion occurred in the presence of 8G5F11 at low pH, indicating that this mAb efficiently blocks the fusion process. We added a paragraph in the result section (lines 373-397): “8G5F11 binding interferes with membrane fusion” and a new Figure (Fig 6: Inhibition of VSV G mediated cell-cell fusion by 8G5F11).

Taken together, our data and those of Munis et al. indicate that fusion is the step that is blocked by the antibody. Accordingly, we decided to remove the passage about the distance created by 8G5F11 binding hindering acess of G to its cellular receptor.

We have added a missing section “Binding assay of VSV G to mAb and Fab 8G5F11 at different pH values” in the Materials and Methods (lines 536–546). Indeed, for the binding assays, antibody (or Fab) and VSV G were separately pre-incubated at the desired pH before complex formation, indicating that **G can bind the antibody or Fab in both the pre- and post-fusion conformations.**

However, as now described in detail in the Materials and Methods section (lines 577-578), for the cryo-EM analysis of the complex, we formed the G-Fab complex at high pH, purified it by SEC, and subsequently i) imaged it directly at pH 8.0 or ii) after decreasing the pH to 5.5 prior to cryo-EM analysis as schematized in the supplementary figure 6C. This strategy allowed us to access the structures of the complexes involving either the pre- or the post-fusion conformation of G. This indicates that Fab 8G5F11 binding does not prevent the conformational change of VSV G when G is in solution.

However, in the context of the viral or cellular surface where spikes are perpendicular to the membrane and probably tightly packed, the situation is different as the antibody movement, in particular the variation of its binding angle from 45 to 135°, cannot be correctly achieved and impedes the structural transition. We illustrated this in the new fig 6C.

All of these observations led us to modify the corresponding paragraph in the discussion, which is now as follows:

*“Regarding the neutralization mechanism, Munis and colleagues [30] showed that the antibody most likely interfered at a post-endocytosis step. In agreement with their observations, we show (i) that the receptor binding site is distinct from the antibody recognition site and (ii) that the antibody inhibits low pH induced cell-cell fusion driven by VSV G. Indeed, even though 8G5F11 allows VSV G conformational change in solution, in the context of the viral or cellular surface where spikes are perpendicular to the membrane and probably tightly packed, the antibody movement, in particular the variation of its binding angle from 45 to 135°, cannot be correctly achieved and impedes the structural transition (Fig. 6C).”*

Reviewer #2: None.

**Part III – Minor Issues: Editorial and Data Presentation Modifications**
Please use this section for editorial suggestions as well as relatively minor modifications of existing data that would enhance clarity.

Reviewer #1: The following clarification and/or additional data would improve the quality of the manuscript:

1. The antibody name 8G5F11 should be mentioned in the Abstract.

We now mention 8G5F11 in the Abstract (line 19).

2. Lines 113-114: Given that pre-to-post structural rearrangement has been proposed to involve transient G dissociation (PMID: 22383886), did the authors see any evidence for de-trimerisation of the VSV-G trimer upon acidification, eg. in micrographs, or in the 2D or 3D classes? A comment on this in the Discussion section would be helpful for readers to understand.

We thank the reviewer for this remark. We could not observe any evidence of G de-trimerization in our dataset following acidification. This is not surprising at pH 5.5, where only post-fusion trimers have previously been detected, both on the virus surface and for the soluble ectodomain in solution. This is more surprising at pH 8.0, where the soluble ectodomain trimer is known to be unstable and prone to dissociation into a monomer. In the present study, the presence of the TMD may help stabilize the pre-fusion trimer. More importantly, it is very likely that monomers known to be highly flexible are not detected in our 2D and 3D analysis.

We have added a comment in the Discussion section (lines 425-430):

“*Although early dissociation of the VSV G trimer has been previously demonstrated, we did not detect such intermediates in our cryoEM analysis. All particles at both pH 8.0 and pH 5.5 appeared trimeric, with no distinct monomer observed in 2D or 3D classifications. This is consistent with the predominance of the post-fusion trimer at pH 5.5. At pH 8.0, the presence of the TMDs may contribute to trimer stabilization. In any case, monomeric intermediates are highly dynamic and thus remain difficult to capture under our experimental conditions*.”

3. Lines 133-136 & 364: It would be of interest to readers if the authors could comment on the angular degree of freedom for the TMD flexibility, for instance, from an estimation using the 2D class averages or masked 3D classification, if possible. This will allow readers an appreciation for the high structural dynamics of the region, while strengthening authors' comparison to the more rigid HCMV and HSV-1 gB, of which the TMDs were resolved structurally.

We thank the reviewer for this suggestion. Due to the absence of clear density for the TMD in our 2D class averages and 3D reconstructions, even after a masking procedure (as already mentioned in the manuscript (line 152)), we cannot provide an estimate of angular degree of freedom for the TMD flexibility. This lack of signal strongly suggests that TMDs are highly dynamic and unlikely to adopt a stable trimeric organization, this is in contrast with the more rigid trimeric TMDs observed for gB in HCMV and HSV-1.

4. Line 154-155, 354-355: VSV NJ G has a polar Y425 instead of nonpolar F425 at the identified CTD hydrophobic cluster. Does this mean this site is dispensable, or compensated by site 423, or the aromatic nature of the side chain is more important than the charge? It's unclear from Figure 2C if it was the side chain or main chain of F425 that interacts with I82. Given that the importance or contribution of this hydrophobic patch was not tested, the authors should mention caveat for their claim.

The substitution of F425 to Y425 in VSV New Jersey G suggests that this position may tolerate some variability, potentially compensated by neighboring hydrophobic residues such as L423, or eventually by the aromatic properties of Y425. We now mention in the Discussion that the functional contribution of this hydrophobic cluster remains to be experimentally validated.

We have added a comment in the Discussion section (lines 412-415):

*“While our results suggest a stabilizing role for the hydrophobic cluster, including F425, the presence of a tyrosine at this position in VSV New Jersey G indicates that some variation toward less hydrophobic residues may be tolerated. However, the precise contribution of this hydrophobic cluster remains to be experimentally validated.”*

5. Lines 378-379: The RABV-G structure determined in reference [38], Ng et al., was solubilised using bOG detergent and subsequently exchanged into amphipol. The authors should correct this, indicating that solubilisation using amphipol, an amphipathic polymer commonly less denaturing and more rigid than detergent, similarly demonstrates the flexibility of TMD regions. Authors should also cite PMID: 35714192 as additional reference which solubilised RABV-G using LMNG.

We thank the reviewer for pointing this out. We have modified the text in accordance, mentioning that in the work of Ng et al. RABV-G was first solubilized using octyl glucoside detergent and then exchanged into amphipol. We also comment on the fact that despite the stabilizing properties of amphipols, the TMD remained unresolved. We also now cite separately the work of Callaway et al., which reports a similar structural analysis of RABV-G solubilized in LMNG. This reinforce our conclusions on the dynamic nature of this region.

We have added in the Discussion section (lines 434-437).

*“Similarly, in a cryo-EM structure of RABV G, solubilized using octyl glucoside that was further exchanged to A8-35 amphipol, the TMDs remained undetected despite the stabilizing effect of amphipols [40] compared with detergent. The same observation was reported for RABV G solubilized in LMNG [42].* *Thus, this raises the possibility that rhabdovirus G TMDs may not associate into a stable trimeric structure. Supporting this hypothesis, tomographic reconstructions of VSV particles, showed no density potentially corresponding to TMDs [39].”*

6. Lines 392-393: Supporting reference(s) should be provided for this hypothesis to support the rationale of this mechanism.

We do not discuss this anymore (see our answer to major comment 2).

7. Lines 491-492: The authors should explain the choice to impose C3 symmetry only on VSV pH8.0 and VSV/Fab pH5.0 reconstructions but not VSV pH5.0 and VSV/Fab pH8.0? Are there any interesting asymmetric features among the three protomers of the molecule in the C1 structures?

C3 symmetry was applied when its improved map quality; it was the case for the reconstructions of VSV G at pH 8.0 and VSV G-Fab at pH 5.5. For the two other reconstructions, C1 gave the best results based on map quality and continuity. We did not observe notable asymmetries in those C1 reconstruction.

8. Minor comments regarding figures:

Fig 1A: The authors should indicate in the legend that the two pinned icons above the VSV-G domain organisational diagram are N-linked glycosylations.

This have been done.

Fig S2, S3, S6, S7: ‘Particles’ spelling mistake in the workflow diagram.

This have been corrected.

Fig S5B & S5D: Non-sensible ladder molecular weight from 25 to 50 back to 37 to 50 to 75.

This have been corrected.

Fig S7: Representative fit of atomic model of G and FAb into density is missing in panel C.

This have been corrected.

Reviewer #2: 1, Figure 1A contains upper and lower panels. For clarity, the figure legend should explicitly define the color representations in both schematics. Additionally, the upper panel of Figure 1A reveals two glycosylation chains. It would be helpful to label the positions of the two glycosylation sites (N163 and N325) in the schematic diagram. Correspondingly, in Figure1B and 1C, the locations of N163 and N325 glycosylation should be labeled in both the pre-fusion and post-fusion structures. This will help readers to understanding the conformational changes of the VSV-G protein.

We thank the reviewer for this suggestion. We have updated Figure 1A legend to define all color representations in both panels (lines 83-86). We also labeled the glycosylation sites N163 and N320 in the schematic (Figure 1A) and in both the pre-fusion and post-fusion cryoEM structures (Figures 1B and 1C) and updated the figure legend accordingly.

2, In Figure 2C, the left panel displays the side chains of L423, F424, F425 from the CTD and I82 from the FD in protomer A, and L105 in protomer B. The right panel shows the side chains of L423, F425 from the CTD and I82 from the FD in protomer A, and L105 and P107 in protomer B. For consistency, the same amino acids should be selected to display their side chains in the two panels. The figure legend should also explain why these residues are selected.

We thank the reviewer for pointing out this discrepancy. We have revised Figure 2C to display the same set of residues in both panels, hydrophobic interactions are indicated only in the close-up for clarity.
We also updated the figure legend with this sentence: “residues displayed as sticks contribute to the stabilizing of the CTD-FD interface in the post-fusion state”.

3, Figures 2C, 2E, 4E, and 4F employ yellow and purple dashed lines with varying thicknesses to represent molecular interactions. The authors should clarify in the figure legends what types of molecular interactions these differently dash lines represent. Alternatively, revise the figures to present more clearly the interaction details.

This have been corrected. Hydrophobic interactions on figures 2C and 2E are indicated by red dashed lines and polar interactions in figures 4E and 4F are in light blue dashed lines. For clarity, interactions are only indicated on the panels showing close-ups.

4, The authors should remove the shadow effects from Figures 2C, 2E, 4E and 4F to improve image readability.

We have removed the shadow effects from Figures 2C, 2E, 4E, and 4F.

5, The BLI assay results in Figures 3B and 3D should include affinity values, do not only provide them in Table S3. In addition, in Figure 4D, the fitted curves show poor agreement with the actual data at 150 nM and 100 nM concentrations, which may affect affinity calculation accuracy.

We thank the reviewer for this comment. We have now added the KD directly in the main figure (3B and 3D) in addition to their presentation in Table S3.

Regarding the fit quality in Figure 3D (for the Fab binding), we agree that the fitted curves at 150 nM and 100 nM slightly deviate from the experimental data during the phase of association.

This likely comes from the rapid on-rate and lower signal amplitude, which affect the precision of the fit at high concentrations. Nonetheless, the global fitting was performed across all concentrations and yielded consistent kinetic parameters (reported in Table S3).

6, In Figure 3, 'mAb' should be replaced by 'Full-length antibody' to match the 'Fab' nomenclature better.

We modified Figure 3 accordingly.

7, In Lines 540-549, the 'VSVΔG-GFP pseudotypes neutralization assay' section only describes pseudotyped virus preparation and titration, lacking the method of neutralization assay.

We thank the reviewer for this remark. We have revised the “VSVΔG-GFP pseudotypes neutralization assay” section to include the description of the neutralization assay. We also added a “VSV**Δ**G-GFP pseudotypes production” section (lines 664-671).

8, Line 289. The heading 'Characterization of 8G5F11 binding site' would be revised as 'Characterization of the key residues for 8G5F11 binding'.

We have modified this heading accordingly. (lines 320).

9, There appears to be a discrepancy in pH values reported for the post-fusion structures: Table S1 lists two structures as "VSV Gfl pH 5.0" and "VSV G-Fab pH 5.0", while the main text (e.g., lines 74 and 78) describes the VSV G post-fusion structure as being determined at pH 5.5.

We thank the reviewer for pointing out this discrepancy. The pH used for post-fusion structure preparations is pH 5.5. This has now been corrected.

10, In the Materials and Methods section, some decimal points are incorrectly formatted as commas. For instance, in line 468. "0,84 Å/pixels" and "49,71 e-/Å") should be revised to “0.84 Å/pixels, 49.71 e-/Å”.

We corrected all decimal commas in the Materials and Methods section and throughout the manuscript to decimal points.
